# Supplementary material for: Artificial light source selection in seaweed production: growth of seaweed and biosynthesis of photosynthetic pigments and soluble protein
Source: PeerJ. 2021 May 11;9:e11351. doi: 10.7717/peerj.11351 (PMC8121058; doi:10.7717/peerj.11351)
Supplement: Supplemental Information 2 [file peerj-09-11351-s002.docx]

**Supplementary Materials**

**
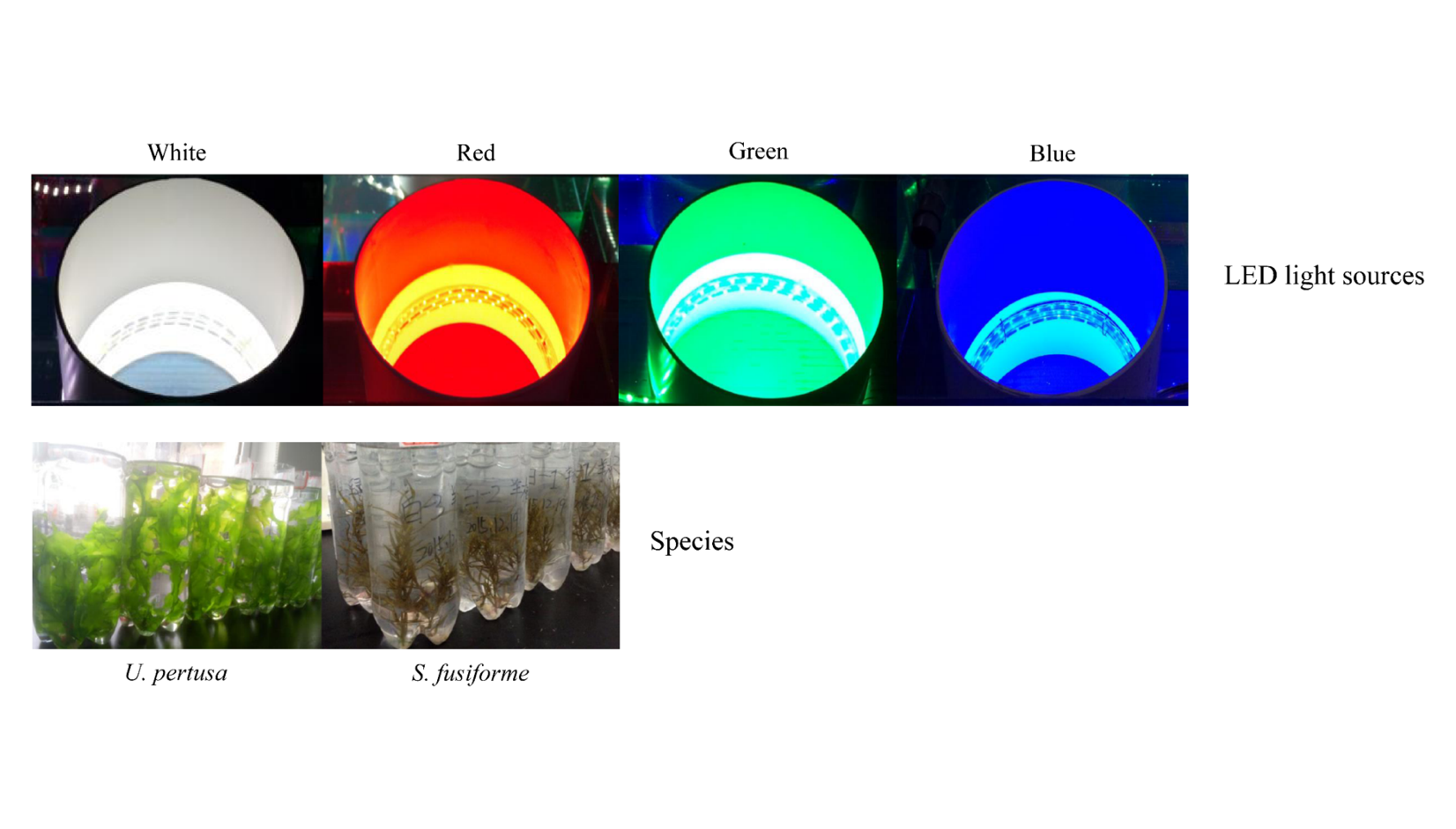
**

**Figure S1. Photos of LED light source settings and experimental species**

**Table S1. Light quality effects on algal biochemical composition.**

| No. | Alga^a^ | Light | Phylum | Index | Effects^b^ | References |
| --- | --- | --- | --- | --- | --- | --- |
| 1 | *Cyclotella nana* | White | Diatom | Growth | B>W>G | (Wallen & Geen, 1971a) |
|  |  | Blue |  | Photosynthesis | B>W>G |  |
|  |  | Green |  | Carbon metabolism | B>W>G |  |
| 2 | *Dunaliella tertiolecta* | White | Chlorophyta | Growth | B>W>G | (Wallen & Geen, 1971a) |
|  |  | Blue |  | Photosynthesis | B>W>G |  |
|  |  | Green |  | Carbon metabolism | B>W>G |  |
| 3 | *Cyclotella nana* | White | Diatom | Total carotene | G>W>B | (Wallen & Geen, 1971b) |
|  |  | Blue |  | Chlorophyll | B>W>G |  |
|  |  | Green |  |  |  |  |
|  |  |  |  | Protein | B>W>G |  |
|  |  |  |  | DNA | B>W>G |  |
|  |  |  |  | RNA | B>W>G |  |
| 4 | *Dunaliella tertiolecta* | White | Chlorophyta | Total carotene | G>W>B | (Wallen & Geen, 1971b) |
|  |  | Blue |  | Chlorophyll | B>W>G |  |
|  |  | Green |  | Protein | B>W>G |  |
|  |  |  |  | DNA | B>W>G |  |
|  |  |  |  | RNA | B>W>G |  |
| 5 | *Isochrysis galbana* | White | Haptophyta | Growth rate | B>W | (Gorai et al., 2014) |
|  | | Blue |  | Chlorophyll *a* | W>B |  |
|  |  |  |  | Carbon content | W>B |  |
| 6 | *Dunaliella salina* | White | Chlorophyta | Growth rate | B>W | (Gorai et al., 2014) |
|  | | Blue |  | Chlorophyll *a* | W>B |  |
|  |  |  |  | Carbon content | W>B |  |
| 7 | *Chaetoceros gracilis*, | White | Bacillariophyta | Growth rate | B>W | (Gorai et al., 2014) |
|  | | Blue |  | Chlorophyll *a* | B>W |  |
|  |  |  |  | Carbon content | B>W |  |
| 8 | *Heterocapsa circularisquama* | White | Myzozoa | Growth rate | B>W | (Gorai et al., 2014) |
|  | | Blue |  | Chlorophyll *a* | W>B |  |
|  |  |  |  | Carbon content | W>B |  |
| 9 | *Porphyra leucosticta* | White | Rhodophyta | PEI^c^ | G>Y>R>W>B | (Korbee, Figueroa & Aguilera, 2005) |
|  |  | Blue |  | Optimal quantum yield | B>W>G>Y>R |  |
|  |  | Green |  | Chlorophyll *a* | G>W>Y>B>R |  |
|  |  | Yellow |  | TINC^d^ | W>B>G>Y>R |  |
|  |  | Red |  | Soluble protein | W>B>G>Y>R |  |
| 10 | *Ulva rigida* | White | Chlorophyta | Chlorophyll *a* | B>W>R>G | (López-Figueroa & Niell, 1990) |
|  |  | Blue |  |  |  |  |
|  |  | Green |  |  |  |  |
|  |  | Red |  |  |  |  |
| 11 | *Corallina elongata* | White | Rhodophyta | Chlorophyll *a* | B>W>R>G | (López-Figueroa & Niell, 1990) |
|  |  | Blue |  | Phycoerythrin |  |  |
|  |  | Green |  | Phycocyanin |  |  |
|  |  | Red |  |  |  |  |
| 12 | *Plocamium cartilagineum (L.)* | White | Rhodophyta | Chlorophyll *a* | B>W>R>G | (López-Figueroa & Niell, 1990) |
|  |  | Blue |  | Phycoerythrin | G>W>B>R |  |
|  |  | Green |  | Phycocyanin | R>W>B>G |  |
|  |  | Red |  |  |  |  |
| 13 | *Porphyra umbilicalis (L.)* | White | Rhodophyta | Chlorophyll *a* | R>W>B>G | (López-Figueroa & Niell, 1990) |
|  |  | Blue |  | Phycoerythrin | G>W>B>R |  |
|  |  | Green |  | Phycocyanin | R>W>B>G |  |
|  |  | Red |  |  |  |  |
| 14 | *Porphyra umbilicalis* | Blue | Rhodophyta | Growth rate | R>B | (Figueroa, Aguilera & Niell, 1995) |
|  |  | Red |  | Chlorophyll *a* | B>R |  |
|  |  |  |  | Phycoerythrin | B>R |  |
|  |  |  |  | Phycocyanin | B>R |  |
|  |  |  |  | Soluble protein | B>R |  |
|  |  |  |  | C | R>B |  |
|  |  |  |  | N | B>R |  |
| 15 | *Gracilaria birdiae* | White | Rhodophyta | Growth rate | G>W>B>R | (Bonomi Barufi, Figueroa & Plastino, 2015) |
|  |  | Blue |  | Chlorophyll *a* | G>B>W>R |  |
|  |  | Green |  | Allphycocyanin | B>W>G>R |  |
|  |  | Red |  | Phycoerythrin | B>G>R>W |  |
|  |  |  |  | Phycocyanin | B>G>R>W |  |

a Microalgae: 1 - 8, macroalgae: 9 - 15

b Example: B>W>G means blue light is more effective than white and green light while white light is superior to green light.

c PEI: Irradiance and photosynthetic effective irradiance.

d TINC: Total intracellular nitrogen content.

Table S2. Light intensity reservation rate of of the white, red, green and blue LED tubes underneath water.

|  |  | White | Red | Green | Blue |
| --- | --- | --- | --- | --- | --- |
| Light intensity  (μmol photons m^-2^ s^-1^) | Air (4 cm)^a^ | 40 | 10 | 25 | 14 |
|  | Water surface (0 cm) | 26 | 6 | 16 | 9 |
|  | Underwater (-4 cm) | 21 | 4 | 14 | 8 |
| Residual energy ratio^b^ | | 95% | 90% | 97% | 97% |

^a^ Distance from water surface

^b^ Residual energy ratio (*RER*): Ratio of residual energy to initial energy for every cm of distance. *RER* was calculated as:

$RER= \sqrt[D]{\frac{E_{B}}{E_{A}}}$ , (S1)

where E_A_ and E_B_ are the energy of the incident light (water surface) and the background light (underwater). D is the absolute distance from A to B.

**Refernences:**

Bonomi Barufi J, Figueroa FL, Plastino EM. 2015. Effects of light quality on reproduction, growth and pigment content of *Gracilaria birdiae* (Rhodophyta: Gracilariales). *Scientia Marina* 79:15–24. DOI: 10.3989/scimar.04049.12A.

Figueroa FL, Aguilera J, Niell FX. 1995. Red and blue light regulation of growth and photosynthetic metabolism in *Porphyra umbilicalis* (Bangiales, Rhodophyta). *European Journal of Phycology* 30:11–18. DOI: 10.1080/09670269500650761.

Gorai T, Katayama T, Obata M, Murata A, Taguchi S. 2014. Low blue light enhances growth rate, light absorption, and photosynthetic characteristics of four marine phytoplankton species. *Journal of Experimental Marine Biology and Ecology* 459:87–95. DOI: https://doi.org/10.1016/j.jembe.2014.05.013.

Korbee N, Figueroa FL, Aguilera J. 2005. Effect of light quality on the accumulation of photosynthetic pigments, proteins and mycosporine-like amino acids in the red alga *Porphyra leucosticta* (Bangiales, Rhodophyta). *Journal of Photochemistry and Photobiology B: Biology* 80:71–78. DOI: https://doi.org/10.1016/j.jphotobiol.2005.03.002.

López-Figueroa F, Niell FX. 1990. Effects of light quality on chlorophyll and biliprotein accumulation in seaweeds. *Marine Biology* 104:321–327. DOI: 10.1007/BF01313274.

Wallen DG, Geen GH. 1971a. Light quality in relation to growth, photosynthetic rates and carbon metabolism in two species of marine plankton algae. *Marine Biology* 10:34–43. DOI: 10.1007/BF02026764.

Wallen DG, Geen GH. 1971b. Light quality and concentration of proteins, RNA, DNA and photosynthetic pigments in two species of marine plankton algae. *Marine Biology* 10:44–51. DOI: 10.1007/BF02026765.
